# Detecting Potentially Adaptive Mutations from the Parallel and Fixed Patterns in SARS-CoV-2 Evolution

**DOI:** 10.3390/v14051087

**Published:** 2022-05-18

**Authors:** Cheng-Yang Ji, Na Han, Ye-Xiao Cheng, Jingzhe Shang, Shenghui Weng, Rong Yang, Hang-Yu Zhou, Aiping Wu

**Affiliations:** 1 Institute of Systems Medicine, Chinese Academy of Medical Sciences, Peking Union Medical College, Beijing 100005, China; chengyang.ji12@alumni.xjtlu.edu.cn (C.-Y.J.); hn@ism.cams.cn (N.H.); yexiao@stu.cpu.edu.cn (Y.-X.C.); sjz@ism.cams.cn (J.S.); wsh@ism.cams.cn (S.W.); yr@ism.cams.cn (R.Y.); 2 Suzhou Institute of Systems Medicine, Suzhou 215123, China; 3 School of Life Science and Technology, China Pharmaceutical University, Nanjing 211100, China

**Keywords:** SARS-CoV-2, COVID-19, mutation, evolution

## Abstract

Early identification of adaptive mutations could provide timely help for the control and prevention of the COVID-19 pandemic. The fast accumulation of SARS-CoV-2 sequencing data provides important support, while also raising a great challenge for the recognition of adaptive mutations. Here, we proposed a computational strategy to detect potentially adaptive mutations from their fixed and parallel patterns in the phylogenetic trajectory. We found that the biological meanings of fixed substitution and parallel mutation are highly complementary, and can reasonably be integrated as a fixed and parallel (paraFix) mutation, to identify potentially adaptive mutations. Tracking the dynamic evolution of SARS-CoV-2, 37 sites in spike protein were identified as having experienced paraFix mutations. Interestingly, 70% (26/37) of them have already been experimentally confirmed as adaptive mutations. Moreover, most of the mutations could be inferred as paraFix mutations one month earlier than when they became regionally dominant. Overall, we believe that the concept of paraFix mutations will help researchers to identify potentially adaptive mutations quickly and accurately, which will provide invaluable clues for disease control and prevention.

## 1. Introduction

**Figure 1:**
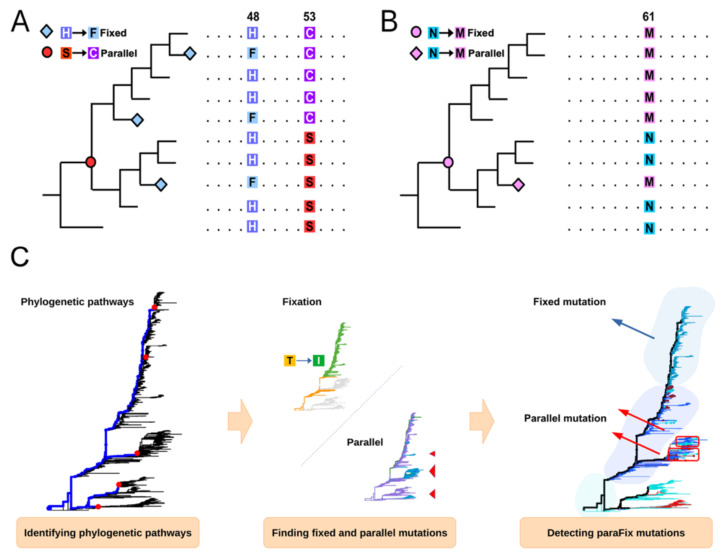
Identification of parallel or/and fixed mutations. (**A**) Fixed mutations refer to the de novo mutations that are fixed in a viral population; for instance, the mutation S → C. Parallel mutations refer to the mutations that appear independently in different phylogenetic pathways; for instance, the parallel mutations H → F appeared in independent phylogenetic pathways. (**B**) The paraFix mutation should have both the fixed and parallel pattern in the phylogenetic tree, similarly to the mutation of N → M. (**C**) Workflow of mutation identification in sitePath tool. Three major steps are included: the phylogenetic pathways are identified (Figure in left, the phylogenetic pathway was labeled blue); the fixed mutations are detected, along each phylogenetic pathway, and the parallel mutations are detected among different phylogenetic pathways (Figure in middle, the tree tips with the same type of amino acid were colored the same); and finally, the paraFix mutations are detected by integrating the fixed and parallel patterns (Figure in the right).

The Coronavirus disease 2019 (COVID-19) pandemic, which is caused by severe acute respiratory syndrome coronavirus 2 (SARS-CoV-2) [1], has imposed a high disease burden worldwide. With one of the longest genomes among all known RNA viruses, SARS-CoV-2 has experienced more than 150,000 mutations in its circulation since its identification in late 2019 (https://bigd.big.ac.cn/ncov/variation/annotation (accessed on 1 April 2022). According to the existing state in the viral population, these mutations could be classified into three categories: random mutation, fixed mutation, and parallel mutation. Of the 150,000 mutations currently identified, most are random mutations, which are the result of random “copying errors” in viral replication or technical sequencing errors [2]. In contrast to random mutations, some SARS-CoV-2 mutations, such as A23403G, which leads to D614G transition in the Spike (S) protein, and A23063T, which leads to N501Y transition in the S protein, became dominant in detected viruses quickly after their initial introduction [3,4]. As these mutations can be inherited and cause independent genetic branches, they appear to “be fixed” in the viral population (Figure 1A), and as such, are defined as fixed mutations. The third type of mutation, parallel mutation or homoplasy, emerges independently in viral genomes of different genetic branches (Figure 1A), such as the E484K/Q/A in the S protein [4].

Despite significant differences, close associations exist among the three types of mutations. Some initially random mutations can act as the “seeds” of fixed or parallel mutations. Moreover, some initially fixed mutations might occur independently in different genetic lineages, as parallel mutations in the later stage, while a few parallel mutations can be fixed after their initial stage. As mutation can change the function or even phenotype of viruses, mutation has various relationships with viral fitness. Most random mutations have a negative or neutral effect on viruses, and these random mutations are often outcompeted by positive selection or epistasis of fixed mutations [5]. However, not all fixed mutations are the result of adaption, and some might be the result of a founder effect and accompany genuine adaptive mutations. Additionally, although parallel mutations might reflect a selective advantage, recombination or sequencing artifacts have the potential to lead to false parallel mutations [6]. Therefore, whether a specific mutation is adaptive or not cannot be inferred solely from its fixation or homoplasy in the viral population.

In this study, we aimed to identify potentially adaptive mutations in SARS-CoV-2, by proposing a new category of mutation, a paraFix mutation (Figure 1B), which is defined as a mutation that has experienced fixation in the viral population and occurs in parallel, in multiple (sub-)lineages. The fixation of one specific mutation might reflect its own selective advantage or a concomitant effect with other adaptive sites. If the fixed mutation in one lineage occurs independently in another lineage, then this mutation has the potential to be an adaptive mutation, instead of being the result of a concomitant effect accompanying the real adaptive mutation. Thus, by searching for paraFix mutations in viral evolution, potentially adaptive mutations can be defined, both quantitatively and accurately.

From the tracking data in one pandemic year, including 27 timepoints during June 2020 and July 2021, 37 sites in S protein were identified as having experienced paraFix mutations. In contrast to the even distribution of fixed-only or parallel-only mutations in S protein, the paraFix mutations were more enriched on the interface of the receptor-binding domain (RBD), N-terminal domain (NTD), or the link region of S1 and S2, which is consistent with the expected functional regions of S protein. Interestingly, 70% (26/37) of the paraFix mutations have already been experimentally confirmed to be beneficial mutations, which conferred the virus with either an enhanced ability for binding to host cells or conferred the virus with the ability to evade antibodies. Moreover, most of the results could be inferred one month before the mutation became regionally dominant. Many paraFix mutations detected in early 2021 have also emerged independently in the new variant of interest (VOI) or variant of concern (VOC) variants, including Lambda, Delta, and even the newly defined Omicron, highlighting the high accuracy of paraFix in predicting potentially adaptive mutations.

## 2. Materials and Methods

### 2.1. Data Acquisition

Phylogenetic trees from 27 time-points between June 2020 and July 2021 were acquired from Nextstrain [7], and the related sequences for each phylogenetic tree were downloaded from Global Initiative on Sharing All Influenza Data (GISAID) [8,9,10]. All the sequences were aligned to SARS-CoV-2 reference genome NC_045512.2 with MAFFT v7.427. In addition, the number of the collected genome sequences along with collection date and the amino acid mutations on each protein product were downloaded from GISAID.

### 2.2. A Brief Review of SitePath

Given the sequence alignment and a rooted phylogenetic tree as input, the R package sitePath [11] first identifies phylogenetic pathways and then finds the parallel and fixed mutation on the pathways (Figure 1C).

The phylogenetic pathways are resolved by finding the commonly shared site polymorphism. By iterating through each site of the input alignment, sequences with the same polymorphism are found. If the number of the sequences is over a pre-defined threshold, their ancestral node on the phylogenetic tree is assumed as the terminal node of a candidate phylogenetic pathway. After all candidate nodes are found, the linked nodes between the tree root and the candidate nodes are collected to represent phylogenetic pathways. Phylogenetic pathways are merged if one is completely overlapped with another.

Parallel and fixed mutations are found by identifying the polymorphism clades on each phylogenetic pathway. By evaluating the polymorphism state of a site, the sequences along a phylogenetic pathway can be grouped in such way that the dominant polymorphism state for adjacent groups is different. To achieve this, sitePath uses a branch-and-bound algorithm to minimize the Shannon entropy value calculated from the polymorphism state across all sequence groups on the phylogenetic pathway. Fixed mutations are the shift of the dominant polymorphism between adjacent clades. Parallel mutations are found by comparing both the fixed and non-fixed mutations among the pathways. However, the number of non-fixed mutations has to exceed a pre-defined threshold to be considered valid.

### 2.3. Detection of ParaFix, Homoplasy, and Episodic Positive Selection Sites

To detect paraFix sites, the translated Spike and N proteins were used as the input for sitePath. The default parameters were used for fixed mutation, and the threshold for parallel mutation was set to 0.1% of the total number of sequences. A site is paraFix if it is recognized as having both parallel and fixed mutations.

To estimate homoplasy, the R package phangorn [12] was used on the whole genome for each subsample dataset, to calculate the consistency index of each genome position. The positions with a consistency index <1 were collected as homoplastic sites and translated to the codon position for the Spike and N protein.

MEME (mixed effects model of evolution) was used for measuring positive selection regarding tree branches/sub-lineages. The coding sequences (CDS) segments for the Spike and N protein were extracted as inputs, because the software only accepts nucleotide sequences and expects the sequence to range from the start codon to the stop codon. According to the Hypothesis Testing using Phylogenies (HyPhy) [13] manual, the default 0.1 *p*-value was used as the threshold for sites with episodic positive selection.

The number of paraFix, homoplasy, and episodic positive selection sites was plotted against the 27 time-points using matplotlib.

### 2.4. Comparison of Performance between SitePath and HyPhy MEME

Although sitePath and HyPhy MEME can both detect episodic selection, their assessment criteria and mechanisms are different in nature. The result of MEME was compared with paraFix sites by drawing a Venn graph and mapping on the Spike protein structure (PDB ID: 7df3). The comparison regarding each time-point was depicted using a dot plot against Spike protein domains. Moreover, the earliest date of the 27 time-points on which the site was recognized as a paraFix was plotted, along with the spatial-temporal distribution of variant-related mutations. The spatial-temporal distribution of the mutations was derived from the genome sequence data provided by GISAID. The Venn graph and other plots were created with matplotlib. The protein structure was rendered by PyMOL.

The scripts for the analysis and plots can be accessed from GitHub (https://github.com/wuaipinglab/SARS-CoV-2_paraFix (accessed on 1 April 2022)).

## 3. Results

### 3.1. Tracking Dynamic Evolutionary Patterns of SARS-CoV-2

**Figure 2:**
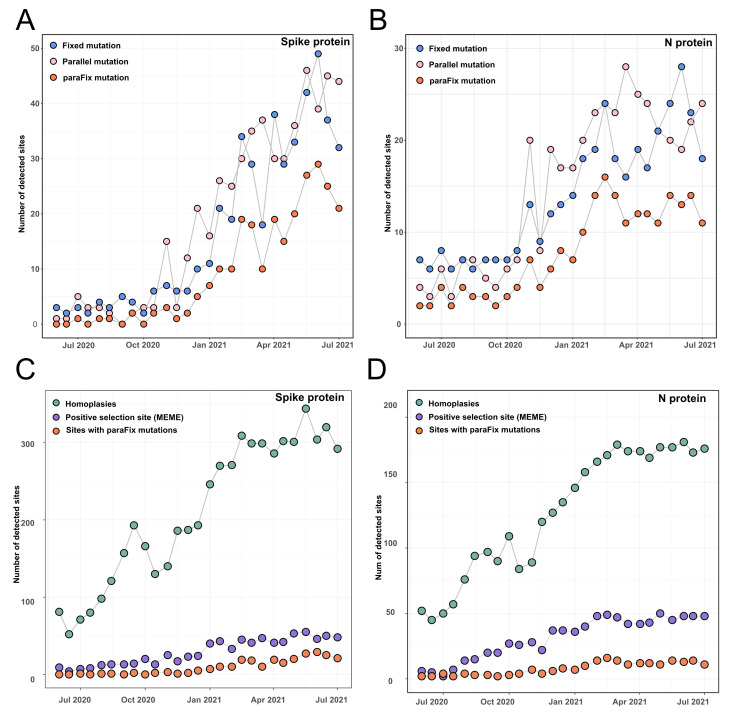
Comparison of identified sites with paraFix mutation for SARS-CoV-2 against positive selection sites and homoplasies. (**A**,**B**) The number of detected sites accumulated with time by considering fixed mutations, parallel mutations, and paraFix mutations in Spike protein (**A**) and N protein (**B**) of SARS-CoV-2. (**C**,**D**) The number of detected sites accumulated with time by considering homoplasies, positive selection sites (inferred with MEME in HyPhy), or paraFix mutation in S protein (**C**) and N protein (**D**).

To detect the dynamic occurrence of potentially adaptive mutations in the SARS-CoV-2 genome, we identified the paraFix mutations in a continuous subsampling dataset, including 27 timepoints of SARS-CoV-2 phylogenetic trees from June 2020 to July 2021 with an interval of 2 weeks. By using an R package called sitePath, developed by us, the fixed mutations and parallel mutations were detected, and then the sites with paraFix mutations were inferred. A total of 265 and 414 sites were identified to be fixed or parallel mutations in the whole SARS-CoV-2 genome, respectively, during the screening period. Whereas only 164 sites were identified to have experienced paraFix mutations (Table S1). Of all 164 sites, 37 were distributed in S protein and 24 were distributed in N protein (Figure 2). Tracing the development of fixed mutations, parallel mutations, and paraFix mutations in S protein and N protein across 27 timepoints, an increase could be observed for the detected sites with time (Figure 2), which may have derived from the increased genetic diversity of SARS-CoV-2 genomes.

### 3.2. Comparison of Sites with ParaFix Mutation, Positive Selection Sites, and Homoplasies

**Figure 3:**
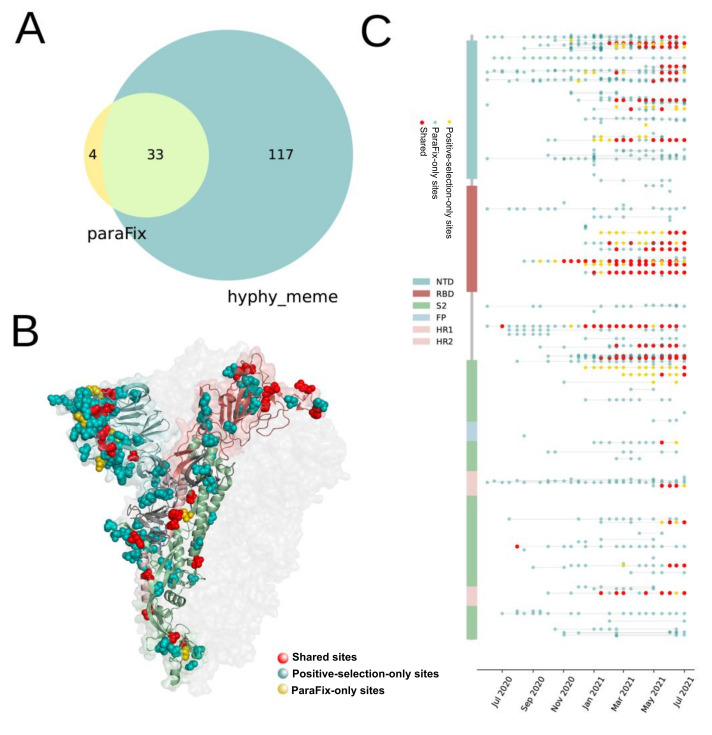
Distribution of sites with parafix mutations and positive selection sites in SARS-CoV-2 S protein (**A**) The number of detected sites with paraFix mutations and positive selection sites. (**B**) The distribution of sites with paraFix mutations and positive selection sites on the structure of S protein (PDB ID: 7df3). (**C**) Time-dependent observation of detected sites with paraFix mutations (Yellow), positive selection sites (blue) and the shared sites in S protein across 27 timepoints, from June 2020 to July 2021.

Positive selection sites and homoplasies are among the two kinds of site that experience putatively natural selections. A comparison of sites that experienced paraFix mutations and the other two types of site were performed. Following suggestions, we introduced HyPhy (MEME) to detect positive selection sites. While the homoplasies were inferred with a tool named phangorn (see Materials and Methods). With this method, the homoplasies were evaluated by calculating the inconsistency of each site in evolution. All sites with paraFix mutations in S and N proteins of SARS-CoV-2 were compared with the results derived from HyPhy (MEME) and phangorn. It was found that the potentially adaptive sites from the three methods showed a similar tendency to increase with time (Figure 2C), despite a significant variation in the number of detected sites. In July 2021, more than 1000 homoplasies were detected by phangorn in S protein, while only 150 sites were identified as positive selection sites by HyPhy (MEME), with 37 paraFix sites by sitePath. Notably, although differing in the number of detected sites, the distribution of the detected homoplasies, positive selection sites, and paraFix sites in S protein was similar (Figure S1). Moreover, all paraFix sites detected using sitePath and positive selection sites by HyPhy (MEME) were subsets of the homoplasies identified by phangorn. Furthermore, 33 of 37 paraFix sites belonged to positive selection sites from HyPhy (MEME) (Figure 3A). Similar results were reached for N and S protein (Figure 2D and Figure S2).

### 3.3. Comparision of Sites with ParaFix Mutaiton and Positive Selective Sites in S Protein

When further comparing the sites derived from HyPhy (MEME) and sites with paraFix mutations (Figure S3B), it was found that a cluster of sites shared by the results of both HyPhy (MEME) and paraFix were mainly distributed on the surface of the receptor binding domain (RBD) of S protein, including 417, 452, 477, 478, 484, 501, and 505. While other clusters of sites shared by both methods were distributed in the linker region of S1 and S2, including 614, 655, 681, 701, and 716 (Figure 3C). All these sites were confirmed to confer the viruses with high fitness (Table 1). However, for four sites inferred only by paraFix mutations (152, 156, 190, and 1117), three of them had already been experimentally confirmed to enhance viral fitness. Thus, most sites (26/37) with paraFix mutations have direct experimental evidence to support their close relationship with viral fitness. Enhancing the infectivity and conferring the virus with antibody escaping ability are the two major contributions of these mutations to the viral fitness of SARS-CoV-2. Although the sites detected only by positive selection were too many and distributed equally on the S protein (Figure 3C), most of these sites could be found on the surface of S protein, indicating their potential association with viral function. The dynamic occurrence of detected paraFix mutations with time showed that they were rare before November 2020, and increased greatly from December 2020, when the newly identified global infections had increased greatly (Figure 3C); while this phenomenon was not obvious for positive selection sites.

### 3.4. ParaFix Sites as Indicators of Potentially Dominant SARS-CoV-2 Variants

**Figure 4:**
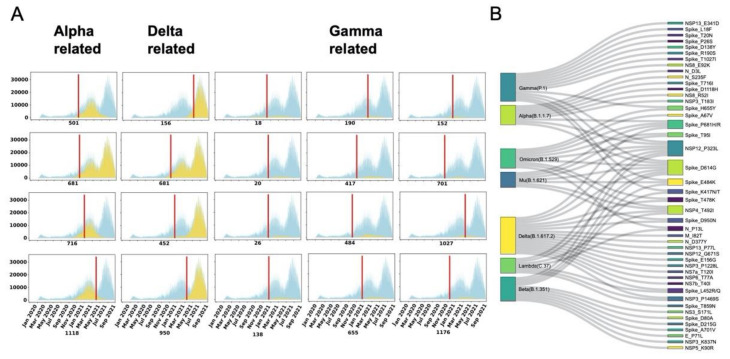
Surveillance of sites with paraFix mutations in different SARS-CoV-2 variants. (**A**) The epidemiological distribution of Alpha-related, Gamma-related, and Delta-related paraFix mutations. The blue area signifies the total isolated infections with time. The yellow area represents the infections with the specific mutation. The red line indicates the first timepoint when this site was recognized as a paraFix mutation. (**B**) The connections between different VOI/VOC variants and the observed paraFix mutations.

A pressing question is whether the detected paraFix mutations could be used as indicators for an outcoming SARS-CoV-2 variant? To answer this question, we plotted the infections detected daily with specific mutations, as shown in Figure 4A. With the tendency to increase with time, infections with paraFix mutations in sites 501, 681, 716, and 1118 were shown to have a close relationship with the outbreak of Alpha lineage viruses. Of these four mutations, N501Y and P681H were detected as paraFix mutations in January 2021, and were the first two detected paraFix mutations in the Alpha lineage. These paraFix mutations were detected approximately 3 months earlier than the maximal infection time of the Alpha linage. Another two paraFix mutations, T716I and D1118H, were detected in the middle and late stages of the breakout of the Alpha lineage. Similarly, for the Delta variant, L452R was detected to be a paraFix mutation in February 2021, 7 months earlier than the maximal infection time of Delta in September 2021. Other mutations, including E156G and D950N, could also be detected as paraFix mutations in the early stage of the Delta pandemic. Regarding the Gamma lineage, a total of nine mutations were detected as paraFix mutations, in which E484K and A701V were first detected, followed by K417N/T, and almost simultaneously, L18F, T20N, P26S, D138Y, R190S, and H655Y. With the exception of T1027I, all eight other mutations were identified as paraFix mutations 2–3 months earlier than the maximal infection time of the Gamma lineage.

We next evaluated the recurrent occurrence of paraFix mutations in VOI and VOC. As shown in Figure 4B, 13 featured mutations of the Gamma lineage belong to paraFix mutations, accounting for 62% of all featured mutations in the Gamma lineage. Similarly, the paraFix mutations also account for 9/17, 11/15, 7/21, 17/26, and 7/18 of the featured mutations in the Alpha, Beta, Mu, Delta, and Lambda lineages, respectively. Recently, the occurrence of the Omicron variant has provided an opportunity to test if the paraFix mutations recurrently occur in future variants; as our results were derived from the dataset until 1 July 2021, when the newly determined variant Omicron was still unobserved. We found that nine paraFix mutations occurred in the Omicron variant, accounting for more than 20% of its featured mutations (Figure 4B). Until now, no evidence has demonstrated that the Omicron variant originated from recombination, and it is possible that these paraFix mutations resulted from de novo mutations.

## 4. Discussion

The accurate and timely detection of adaptive mutations in viral evolution is an important topic, especially during the ongoing SARS-CoV-2 pandemic. Given the time- and labor-consuming nature of experimental detection, computational methods were developed to predict potentially adaptive mutations. For instance, the homoplasy-based method was developed for inferring potentially adaptive mutations, supposing that viruses under similar selective pressures would evolve parallelly and independently. However, use of the homoplasy-based method in large-scale SARS-CoV-2 genomes led to too many homoplasies being detected, most of which were confirmed to be under neutral or negative selection. Calculating the dN/dS is another commonly used method, which can infer the positive selection (dN/dS > 1) or negative selection (dN/dS < 1) of viral sites. However, the limitation of applying the dN/dS ratio in SARS-CoV-2 is that SARS-CoV-2 has evolved many co-evolved synonymous and nonsynonymous mutations, which might hamper the calculation and lead to bias.

We developed a strategy to detect the potentially adaptive mutations from their fixed or/and parallel patterns in the phylogenetic trajectory. A fixed mutation is a novel mutation that has almost totally replaced the parental amino acid or nucleotide since its occurrence in the viral population. The dominance of fixed mutations in the progeny virus potentially reflects the priority of the fixed amino acid/nucleotide toward the replaced one. However, we must point out that the founder effect may also lead to the fixation of mutations, which would introduce error when identifying beneficial mutations based on their fixation. The concept of parallel mutation in our method is similar to that of homoplasy. However, the difference is that the detection of parallel mutations in our method is based on the continuous phylogenetic pathway. Although a mutation that occurs in multiple phylogenetic pathways independently may indicate a potential advantage of fitness, we cannot exclude the possibility that it is a random mutation. Therefore, by integrating parallel mutation and fixed substitution to define the paraFix mutation, we propose that the biological meanings of these two types of mutations are highly complementary, and can reasonably be used to identify potentially adaptive mutations.

By screening 27 timepoints during the evolution of SARS-CoV-2, 164 sites in viral genomes were detected as experiencing paraFix mutations. As expected, under the strongest selective pressure, the S protein was detected as having the most frequent paraFix substitutions (37/164). Compared with the sites inferred from HyPhy, the inferred sites with paraFix mutations might have a higher correlation with viral fitness. Notably, most of the sites with paraFix mutations belong to positive selection sites. Some positive selection sites were distributed closely in the structure of S protein that formed several small “hot regions”. Interestingly, most paraFix sites were located in the “hot regions” formed by positive selection sites, suggesting that these paraFix mutations demonstrated a more competitive nature in viral adaptation, which can be partly proven by three pieces of evidence. First, we found that almost all of the sites with paraFix mutations in the S protein occurred either in the link region of S1 and S2 or on the interaction surface of the RBD, which are subjected to strong selective pressure in viral adaptation. Second, most of our detected paraFix mutations had been previously confirmed during experiments investigating their promotion of viral fitness (Table 1). For example, D614G was identified as increasing viral infectivity by forming a more open conformation, N501Y and S477N were confirmed to enhance viral binding to host cells, and E484K was proven to help the virus escape natural immunity (Table 1). Third, the detected potentially adaptive mutations were shared by multiple VOI or VOC. Interestingly, even in the newly defined VOC Omicron, nine of the 45 (20%) featured mutations are paraFix mutations.

Overall, we believe that the concept of paraFix mutations will help researchers to identify potentially adaptive mutations quickly and accurately, which will provide invaluable clues for disease control and prevention.

## Figures and Tables

**Table 1:** Experimental evidence for the identified paraFix mutations of SARS-CoV-2.

Mutation	Functions	References
L5F	Enhanced infectivity with D614G, but decreased infectivity without D614G	[14]
S12F	Indirectly contributes to antibody escape	[14]
L18F	1. Escape of antibody S2L28 2. L18F, T20N, and D138Y contributed to the loss of activity of samples 2–17 and samples 4–19	[15,16]
T20N	L18F, T20N, and D138Y contributed to the loss of activity of samples 2–17 and samples 4–19	[16]
P26S	Partially accounted for the loss of activity of samples 4–18	[16]
A67V	-	-
D80A	1. Slightly reduced the antibody neutralization of S2L28 2. High resistance to convalescent plasma of P6 and high sensitization to P18	[16,17]
T95I	T95I substitution occurs outside the antigenic supersite and is unlikely to significantly contribute to immune evasion	[18]
S98F	-	-
D138Y	L18F, T20N, and D138Y contributed to the loss of activity of samples 2–17 and samples 4–19	[16]
W152C	1. Both R190S and W152C impair binding of samples 5–7 by altering the local conformation of NTD loops 2. R190S and W152C each attenuate the pseudovirus half-maximal inhibitory concentration (IC50) by 6.1- and 18.9-fold, respectively	[19]
E156G	Residues 156–158 participate in the supersite β-hairpin and their mutation/deletion in the B.1.617.2 NTD lead to striking structural remodeling	[18]
F157L	Residues 156–158 participate in the supersite β-hairpin and their mutation/deletion in the B.1.617.2 NTD lead to striking structural remodeling	[18]
R190S	1. Both R190S and W152C impair binding of samples 5–7 by altering the local conformation of NTD loops 2. R190S and W152C each attenuate the pseudovirus half-maximal inhibitory concentration (IC50) by 6.1- and 18.9-fold, respectively 3. L18F, T20N, D138Y, and R190S together resulted in the loss of activity of samples 5–7	[16,19]
D215G	High resistance to convalescent plasma of P6, P8, P11, P13, P15, P17	[17]
A222V	Not strong for antibody escape	[15,20]
K417N/T	K417N seems to eliminate neutralization by co-occurrence with E484K	[21,22]
N439K	N439K increases the spike affinity for hACE2; viral fitness and disease are unchanged, N439K confers resistance to several mAbs and escapes some polyclonal responses	[23]
L452R/Q	The L452R mutation reduces neutralization mediated by some clinical mAbs, such as bamlanivimab (LY-CoV555) and regdanvimab (CT-P59), due to steric alteration of the antigenic site, which is incompatible with binding	[18,24]
S477N	No impact was observed on either mAb with the S477N or K537R variants	[25]
T478K	Requires further investigation	[18,26]
E484K/Q	Antibody escape	[27]
N501Y/T	Enhances SARS-CoV-2 infection and transmission	[28]
D614G	Increases viral infectivity	[29]
H655Y	Confers SARS-CoV-2 with competence priority	[30]
Q677H	Increases viral infectivity and syncytium formation, and enhances resistance to neutralization for VOCs	[31]
P681H/R	Increases viral infectivity	[32,33]
A701V	High resistance to convalescent plasma of P2 and P11	[17]
T716I	High resistance to convalescent plasma of P6, P11, and P13	[17]
T732A	-	-
T859N	No influence on infectivity or vaccine-induced neutralization	[34]
D950N	D950N substitutions are part of epitopes known to be recognized by neutralizing Abs	[18]
T1027I	No major changes in S2, and V1176F is in a disordered region	[35]
A1078S	-	-
T1117I	-	-
D1118H	1. High resistance to convalescent plasma of P6, P8, P11, and P13. 2. Decreases viral fitness in hamster	[17,28]
V1176F	V1176F is in a disordered region.	[35]

## Data Availability

All the scripts can be found at https://github.com/wuaipinglab/SARS-CoV-2_paraFix (accessed on 1 April 2022).

## Supplementary Materials

The following supporting information can be downloaded at: https://www.mdpi.com/article/10.3390/v14051087/s1, Figure S1: The detected homoplasis (left), positive selection sites (middle) and sites experienced paraFix (right) in S protein; Figure S2: The distribution of detected adaptive related sites in N protein; Table S1: Detected sites that have experienced paraFix mutations.
