# Supplementary material for: Detecting Potentially Adaptive Mutations from the Parallel and Fixed Patterns in SARS-CoV-2 Evolution
Source: Viruses. 2022 May 18;14(5):1087. doi: 10.3390/v14051087 (PMC9147038; doi:10.3390/v14051087)
Supplement: Supplementary file 1 [file viruses-14-01087-s001.zip › Supporting_Information_viruses_revise.pdf]

Supplementary Information for

Detecting potentially adaptive mutations from the parallel and fixed patterns in SARS-CoV-2 evolution

Cheng-Yang Ji, Na Han, Ye-Xiao Cheng, Jingzhe Shang, Shenghui Weng, Rong Yang, Hang-Yu Zhou and Aiping Wu

Corresponding to: Aiping Wu (wap@ism.cams.cn) and Hang-Yu Zhou (zhy@ism.cams.cn)

This PDF file includes:

Figs. S1, S2
Captions for Table S1

Other Supplementary Materials for this manuscript include the following:

Table S1

Table S1 Detected sites that have experienced paraFix mutations.

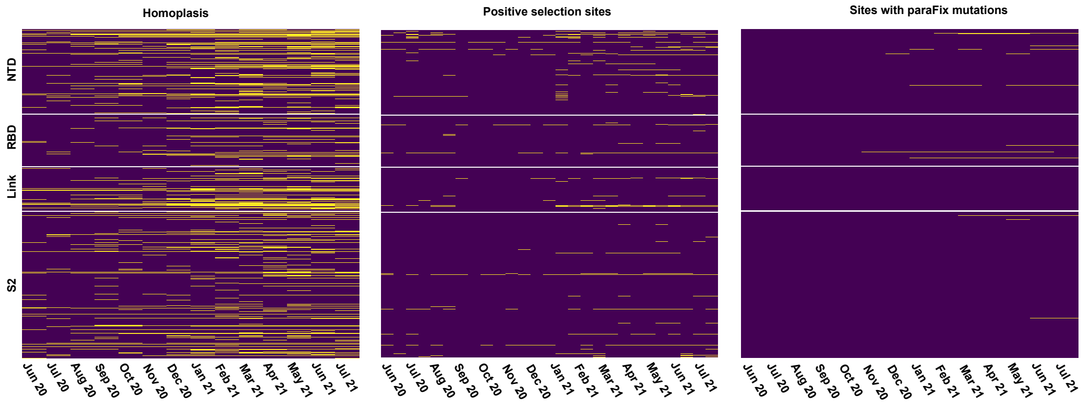

Fig. S1 The detected homoplasis (left), positive selection sites (middle) and sites experienced paraFix (right) in S protein.

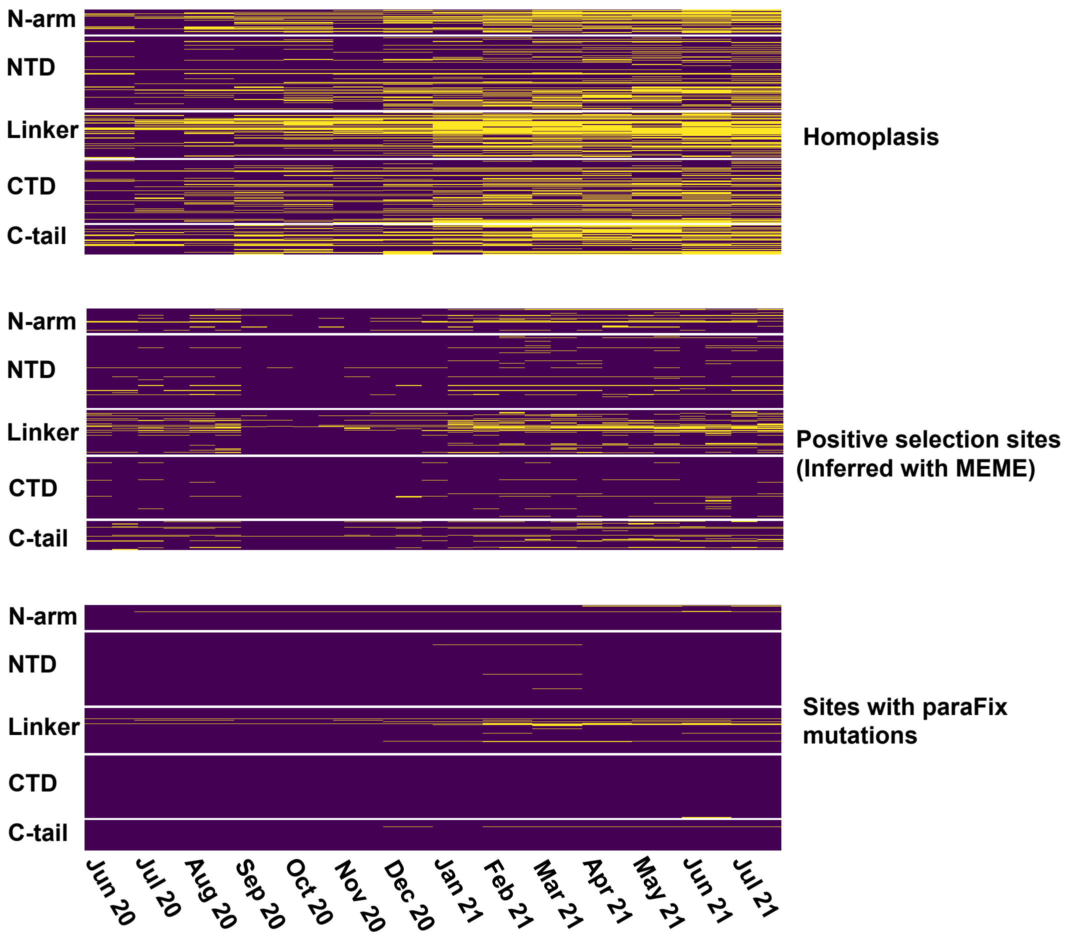

Fig.S2 The distribution of detected adaptative related sites in N protein.
